# High-quality photonic crystals with a nearly complete band gap obtained by direct inversion of woodpile templates with titanium dioxide

**DOI:** 10.1038/srep21818

**Published:** 2016-02-25

**Authors:** Catherine Marichy, Nicolas Muller, Luis S. Froufe-Pérez, Frank Scheffold

**Affiliations:** 1 Department of Physics, University of Fribourg, Chemin du Musée 3, CH-1700, Fribourg, Switzerland; 2 Laboratoire des Multimatériaux et Interfaces - UMR 5615 CNRS / UCBL, Université Claude Bernard Lyon 1, 22 avenue Gaston Berger, 69622 Villeurbanne Cedex, France

## Abstract

Photonic crystal materials are based on a periodic modulation of the dielectric constant on length scales comparable to the wavelength of light. These materials can exhibit photonic band gaps; frequency regions for which the propagation of electromagnetic radiation is forbidden due to the depletion of the density of states. In order to exhibit a full band gap, 3D PCs must present a threshold refractive index contrast that depends on the crystal structure. In the case of the so-called woodpile photonic crystals this threshold is comparably low, approximately 1.9 for the direct structure. Therefore direct or inverted woodpiles made of high refractive index materials like silicon, germanium or titanium dioxide are sought after. Here we show that, by combining multiphoton lithography and atomic layer deposition, we can achieve a direct inversion of polymer templates into TiO_2_ based photonic crystals. The obtained structures show remarkable optical properties in the near-infrared region with almost perfect specular reflectance, a transmission dip close to the detection limit and a Bragg length comparable to the lattice constant.

Even though three-dimensional Photonic Crystals (PCs), made of high index materials such as titanium dioxide^1^,^2^ and silicon^3^, have been fabricated for instance by successive sputtering and electron beam patterning or from opal templates^4^,^5^,^6^, the infiltration of a lithographically structured polymer template appears to be a particularly effective strategy for obtaining large three dimensional PCs^7^,^8^ and for embedding functional subunits for application in optical circuits or devices^9^,^10^,^11^. Tétreault *et al.* reported the first silicon replica of such a woodpile template using the silicon double inversion technique^12^. A SiO_2_ inverse woodpile PC was used as an intermediate structure that was subsequently infiltrated with silicon by employing high temperature chemical vapor deposition (CVD). Later, the same group reported on the fabrication of silicon inverse woodpile PCs^7^. In this case, a SiO_2_ shell was deposited around the polymer rods of the template to preserve the log-pile structure during the silicon deposition process. By these and related approaches, silicon hollow-rod woodpile PCs^13^, waveguides^11^, hyperunifrom^14^ and complete band gap PCs at telecommunication wavelengths around 1.54 *μ*m^8^ have been obtained. Another well-known high refractive index material that has been widely used in the past is titania (TiO_2_, titanium dioxide)^1^,^6^,^15^. The advantages of titania, compared to silicon, are its transparency in the visible to mid-infrared region as well as the possibility to chemically wet-process. In addition, titania can be deposited at moderate temperatures around or below 100 °C. This makes titania a particularly well-suited material to infiltrate three-dimensional polymeric scaffolds directly without the need for any additional infiltration steps which tend to lead to structural deterioration.

In this work we present the fabrication, structural and optical characterization of titania hollow-channel and inverse woodpile PCs. Our work reports two main achievements in the field. First, we report on the fabrication of high-index woodpile PCs in a single infiltration step process. This facilitates, as we will show, the fabrication of structures and yields an outstanding structural integrity and surface quality. Second, we present a direct measurement of the Bragg length L_B_ in our high-refractive-index PCs. The Bragg length is a key parameter for the characterization of photonic band gap materials as it sets the length scale an evanescent wave can penetrate into a PC material. As such it also sets the lower bound for the design of integrated structural features such as bends and cavities. The latter have to be separated by several Bragg lengths in order to prevent cross-talking or tunneling. Despite the importance of the Bragg length very little experimental data on high-refractive-index materials has been reported to date in the literature. A number of studies have the Bragg length for the case of low-index materials in the vicinity of the corresponding pseudo-gaps^16^,^17^,^18^. Here we demonstrate that owing to the precision of our materials fabrication process we can reproducibly fabricate high-refractive-index photonic crystals with different layer thicknesses ranging from 12 to 32 layers (one unit cell is composed of four layers). By measuring the transmission dip minimum as a function of the thickness of the PC layer, we directly determine the Bragg length L_B_ for one given lattice orientation.

Photonic titanium dioxide structures have already been realized in the past by single or double inversion/infiltration methods of polymer templates by sol-gel wet processing^19^,^20^,^21^,^22^ sputtering^23^ and via atomic layer deposition (ALD)^15^,^24^,^25^,^26^,^27^,^28^,^29^,^30^,^31^. ALD provides control of the film thickness at the atomic scale already at moderate temperatures^26^,^27^. Consequently, ALD also allows the elaboration of a thin protective shell around thermally unstable polymer templates for example by depositing Al_2_O_3_^32^,^33^ or TiO_2_^34^. In a subsequent step such stable hybrids can be infiltrated with silicon using thermal CVD as shown for the case of polymer woodpiles^32^,^33^ and hyperuniform network structures^34^. While woodpile PCs made of a polymer core and of inorganic shell have also been obtained using TiO_2_ ALD coating^24^,^25^, a direct full inversion of polymer woodpile PCs with ALD of TiO_2_ has not been reported so far to our knowledge. Graugnard *et al.*^35^ reported TiO_2_ replica of holographically defined polymer templates by double inversion techniques using ALD. After inversion of the polymer structure into Al_2_O_3_ using a low temperature ALD process, a second ALD infiltration with TiO_2_ was performed. The 3D PC consisted of 23 layers and the successful infiltration by ALD through several layers was demonstrated. Recently, direct TiO_2_ woodpile PCs displaying a full PBG were obtained by a similar approach using an intermediate ALD ZnO step^15^. Despite the comprehensive work that has already been performed on TiO_2_ woodpile-based 3D PCs, very few of these studies show evidence for the theoretically predicted near-zero transmission dips as well as near 100% reflection peaks. This suggests that the material properties, such as mean refractive index and structural integrity, are not sufficiently close to the assumptions made in theoretical band structure calculations.

## Results

### Material fabrication and characterization

In the present work we show that direct infiltration with ALD and subsequent removal of the polymer template by calcination result in high-quality photonic materials that allow a near-quantitative comparison between calculated and experimental spectra of TiO_2_ hollow-rod and inverse woodpile PCs. Combining multiphoton lithography, also known as direct laser writing (DLW), with atomic layer deposition, we successfully invert polymer woodpile templates into TiO_2_. Due to the high conformity and the uniformity of the infiltration process, high-quality TiO_2_ inverse woodpiles are obtained. The latter display remarkable optical properties in the near-infrared region (1.4–1.7 *μ*m) with a reflectance peak close to 100% and a transmittance dip close to the detection limit.

**Figure 1:**
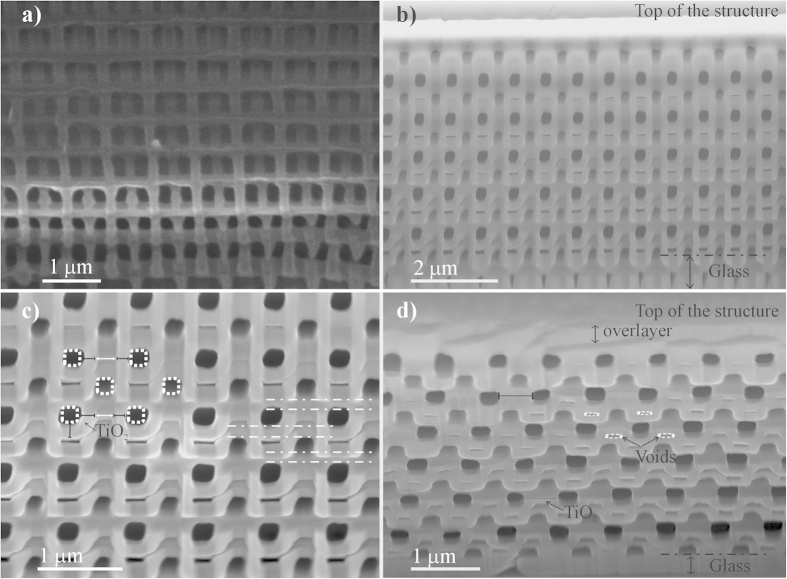
SEM images of (**a**) polymer, (**b,c**) partially TiO_2_ infiltrated woodpile structures, and (**d**) nearly completely infiltrated structures obtained after 1500 and 4500 ALD cycles, respectively. The polymer template has been removed by calcination (**b–d**). Cross-sections are cut using a focus ion beam with viewing angles (**a**) 40° (**b,c**) 45° and (**d**) 25°. In the enlarged image (**c**) of the partially TiO_2_ infiltrated woodpile shown in (**b**), the dotted circles and dashed lines represent the rod positions in the initial polymer template; the TiO_2_ is indicated by black lines while the interspaces between two TiO_2_ hollow channels are highlighted by the full white lines. The image in (**d**) reveals the presence of residual air voids (dotted circles) inside the inverted titanium dioxide structure. The metal oxide over-layer on the top of the structure is also shown.

**Figure 2:**
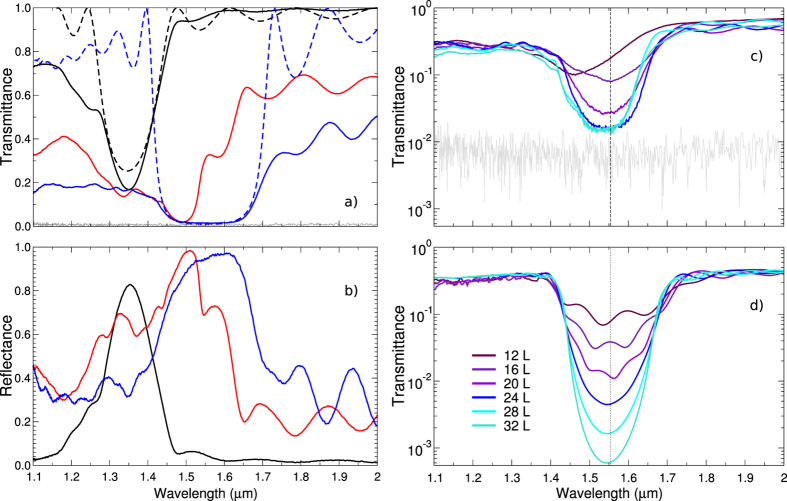
(**a**) Transmittance and (**b**) reflectance spectra from the polymer template as obtained by DLW (black full line), the partially infiltrated TiO_2_ hollow-rod structures (red full line) and the TiO_2_ inverted structure (blue full line). Calculated transmittance spectra at normal incidence are shown as dashed lines. (**c**) Measured transmittance spectra in log-plot representation for TiO_2_ inverse woodpiles with different numbers of layers (see legend). The gray line indicate the background noise signal recorded on a gold mirror as a beam block. (**d**) Normal incidence calculated spectra with a gap center wavelength of 1.54 *μ*m (dashed vertical line).

The polymer woodpile templates, shown in Fig. 1a, are written using DLW and the novel dip-in method combined with a shaded-ring filter using the commercial ‘Photonic Professional’ platform (Nanoscribe GmbH, Germany). This particular combination of techniques permits to fabricate rods with a low aspect ratio and thus leads to structures with a unprecedented quality. Indeed, while by regular DLW stop bands with less than 60% in reflection are typically measured with unpolarized light^7^,^36^, the initial PCs presented here exhibit a band gap of 80–85% in reflection and a dip in transmission lower than 20% (Fig. 2). The templates present on average a lateral rod spacing of ∼750 nm and a rod diameter of 235 nm yielding stop bands around 1.2–1.35 *μ*m. Only small variations are observed between different fabrication runs. Massive polymer walls of 10 *μ*m thickness are lithographically placed around the woodpiles in order to increase their mechanical stability along the development and infiltration processes. Structures made of 24 layers are partially and completely infiltrated with TiO_2_ by ALD at moderate deposition temperatures. The degree of infiltration is controlled by the number of applied cycles. We subsequently remove the polymer by high temperature calcination and transform the as-deposited amorphous titanium dioxide into its denser anatase phase. Incomplete infiltration with titania thus leads to structures composed of hollow titania rods and for longer infiltration times we obtain (almost) completely inverted titania woodpile PCs. The infiltration, occurring in a conformal manner, unavoidable small voids are formed due to clogging that prevents the precursors to diffuse further in the final stages of the infiltration process. In Fig. 1b–d, scanning electron microscope (SEM) images recorded from cross-sections of both types of TiO_2_ structures reveal their high-quality and the good homogeneity of the deposition from top to bottom without significant variations of the thickness. The bright contrast indicates the TiO_2_ and the dark contrast the voids left over by the degraded polymer. One should note that the writing process is started in a virtual depth of 2 layers inside the glass substrate (shown in Fig. 1b,d) to guarantee a continuous laser writing process along the axial direction which is necessary to ensure the adhesion of the polymer template to the substrate. Woodpile PCs composed of hollow rods are obtained by partial infiltration (stopped after 1500 cycles). In Fig. 1c, a titania layer of finite thickness (indicated by black lines) is grown around the polymer rods (their initial positions are pointed out by dotted white circles). Moreover the quincunx distribution of out- and in-plane layers is clearly visible. The in-plane layers are highlighted by dotted white lines. Horizontal interspaces between two neighboring shells of an out of plane layer can be distinguished and indicate the absence of coalescence of the shells due to partial infiltration. In the case of the fully infiltrated TiO_2_ inverse woodpile no such interspaces are noted and an over-layer is visible on top of the structure (Fig. 1d).

Atomic force microscopy (AFM) measurements (Supplementary Fig. 1) reveal the smoothness of the surface with variations of the root mean square roughness (RMS) along a rod on the order of 2–3 nm only which is a sign of the high conformity of the coating. In the AFM images of the hollow rod woodpile PCs the first layer perpendicular to the top one is visible while only the top rods are noted for the TiO_2_ inverse structure, confirming the partial and full infiltration. Spectroscopy data in the mid-IR confirm the removal of the polymer during the high temperature annealing step, and the densification of the TiO_2_ (Supplementary Fig. 2).

### Near infrared spectroscopy

The optical properties of the structures are characterized by means of a Fourier transform infrared spectrometer in combination with a grazing angle objective. We measure the near-infrared reflectance and transmittance along incident angles which are contained in a cone ranging from 10° up to 30° with respect to the surface normal. The normalization of the transmittance and reflectance are performed on a bare glass substrate and a gold mirror, respectively. Figure 2 shows the optical response of polymer, TiO_2_ hollow-rod and TiO_2_ inverse woodpile PCs in transmittance and in reflectance. For the polymer woodpile PCs (full black lines) we observe a strong and narrow transmission dip as well as a strong reflection peak at 1.35 *μ*m. After both partial and complete infiltration and polymer removal, a redshift as well as a broadening of the dip and peak are noticed. For the case of completely infiltrated structures, the transmittance drops below 2% and the reflectance reaches almost 100%. The inverse woodpile PCs (blue full line) show a robust stop band centered at 1.54 *μ*m which is 18.5% wide, while the band gap at 1.5 *μ*m of the hollow-rod woodpile is less well defined even though still relatively broad (see also Supplementary Fig. 4). As visible in Fig. 2a, the transmission characteristics of the TiO_2_ hollow rod-structure (red full line) are not as well developed as for the inverse woodpile. For both titanium dioxide PCs, Fabry-Pérot fringes are visible, highlighting the good quality of the infiltration and the integrity of the structures during the entire fabrication process. Indeed, only high-quality PCs with homogeneous optical properties along the z-axis can exhibit such features.

### Comparison to numerical calculations

For a comparison with numerics we calculate the total transmission and reflection spectra using Finite Difference Time Domain (FDTD) methods^37^. To obtain suitable parameters for modeling the woodpile structure, photonic band structure calculations were first carried out in order to fit the 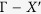 stop gap center and width (corresponding to normal incidence) to the measured spectra. As shown in Fig. 2, the position of the measured band gap, its depth, and the Fabry-Pérot oscillations are nearly quantitatively reproduced by the calculations. Outside the gaps we observe deviations. For small wavelengths we can attribute these deviations to diffraction and reflections at the interface. Diffraction from Bragg planes redirects incident light to well defined Bragg angles^38^. Initially these angles are close to the backscattering direction but gradually they move towards angles as the wavelength is lowered. In the simulations we sum up all transmitted and reflected power and thus 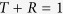, while in the experiments the detection is limited to a finite range of detecting angles as explained in the methods section and therefore 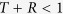 outside the gap. For the higher wavelengths the differences between theory and experiment can be explained by interfacial reflections and scattering by residual lattice distortions^39^.

In order to investigate the influence of the structure height on the stop band, we fabricate woodpile PCs templates of different heights with a number of layers ranging from 12 to 32. Subsequently, the templates are fully infiltrated with ALD TiO_2_ and calcinated. The number of ALD cycles has been reduced to 3500 in order to significantly decrease the cap -layer. The transmittance spectra of the obtained inverse woodpile PCs are presented in Fig. 2c. A minimum of 24 layers is required to reach a transmittance as low as 1–2%. A further increase of the height of the PC does not reduce the apparent depth of the gap. This observation can be explained by residual fabrication errors and distortions during development, infiltration and polymer degradation. However, the contribution of these imperfections cannot easily be quantified since the FTIR spectroscopy as well as the normalization can also lead to errors that may even become dominant for transmission coefficients on the order of one percent as shown in Fig. 2c,d.

**Figure 3:**
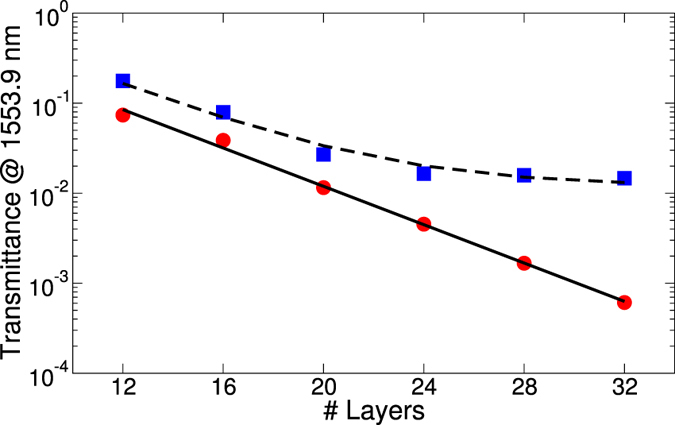
Calculated (circles) and measured (squares) transmittance at a wavelength lying in the 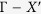 bandgap, center wavelength of   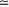 1.54 *μ*m. The full line shows the best fit of the simulated data to an exponential 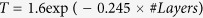. The dashed line shows an exponential decay with a slightly adjusted prefactor using same decay length as in the calculations leading to excellent agreement with the experimental data 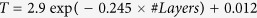. A small baseline value is added for a best fit in order to take the instrument background noise and other imperfections of the measurement into account.

**Figure 4:**
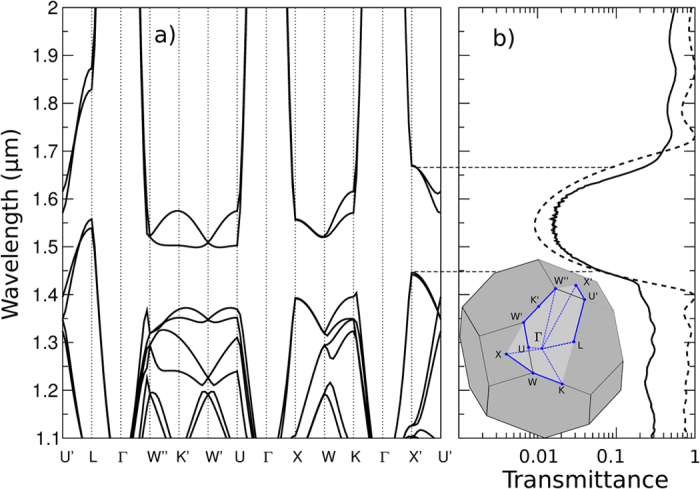
(**a**) Calculated photonic band diagram for the inverted woodpile PC shown in Fig. 1d. The parameters of the band structure calculation are taken from the comparison to the experimental data as shown in panel (**b**). In order to take the effect of voids in the structure into account, a reduced effective refractive index was determined to n_eff_ = 2.12 instead of n = 2.4 for bulk TiO_2_-anatase. (**b**) Calculated normal incidence transmission spectrum (angle integrated at the output) for an inverted woodpile of 24 layers (dashed line) and the comparison to the measured spectrum (full line). The inset shows the geometry of the first Brillouin zone, a slightly stretched FCC lattice, as well as the points of high symmetry.

Next we compare the experimental results to FDTD calculations at normal incidence (Fig. 3). Fitting the decay of T_Gap_ (L) from FDTD calculations at a wavelength λ = 1553.9 nm with an exponential we again find a Bragg length of L_B_ ∼ 4 layers which corresponds to the thickness of one unit cell. We define the Bragg length L_B_ as the transmittance decay length. Using this theoretically obtained Bragg length and a baseline transmittance of 1.2% an excellent fit to the experimental data is obtained. The difference in the prefactor of the exponential can be attributed to the subtle differences in the surface termination between the actual structure and the simulated one. The experimental, quantitative extraction of the Bragg length of a photonic material with a nearly complete bandgap is a remarkable result of this study. Previous measurements of the Bragg length have been reported for low-index polystyrene opal PCs^16^,^17^,^18^ and for silicon inverse woodpile PCs^40^. In the latter case a Bragg attenuation length of ∼7 layers was found. It is telling that this value reported previously is actually higher than the one measured in the present study despite the nominally lower refractive index of bulk TiO_2_. We thus believe that the small Bragg length observed in our study is yet another proof for the excellent quality of our titania PC. Finally, in Fig. 4, we show the calculated full band diagram obtained for the effective material properties extracted from the comparison of theory and experiment in transmission and reflection. In order to take the effect of voids in the structure into account a reduced effective refractive index n_eff_ = 2.12 is used for the calculations instead of n = 2.4 for bulk TiO_2_-anatase in the near infrared. The band structure displays a nearly complete band gap with a center wavelength of 1.54 *μ*m. Only around the *U*′ − *L* − Γ directions, for a narrow solid angle, bands slightly overlap. Figure 4b shows again the excellent agreement between experiment and numerical FDTD calculations for the case of normal incidence transmission.

## Discussion

We were able to invert polymeric woodpile PCs into titania by using a single step infiltration approach. The high-quality and homogeneity of the as-infiltrated structures is confirmed by SEM and the presence of Fabry-Pérot fringes in the optical response. The PC materials fabricated exhibit very small values for transmission as well as a near 100% reflection peak at a wavelength of 1.54 *μ*m and a gap width of 18.5%. The results are obtained reproducibly and thus provide a robust platform for the future implementation of PC cavities and waveguiding. Thin air channels inside the inverted structure can be attributed to the conformal nature intrinsic to ALD processes and can be taken into account for FDTD modeling by using a reduced effective refractive index of the TiO_2_. The measured spectra are in good agreement with band diagram calculations and transport simulations. We can clearly distinguish between a partially and completely infiltrated case. Furthermore, the experimental method presented here is highly flexible and can be used to invert arbitrary two or three-dimensional polymeric structures into titania.

## Methods

### Direct-Laser Writing (DLW)

The woodpile photonic crystals were fabricated using a commercially available setup (Photonic Professional from Nanoscribe GmbH) in combination with the novel available Dip-In technique and a shaded ring filter. Structures were written on glass and CaF_2_ (Crystan, UK) substrates by *dipping* the objective directly inside a liquid negative-tone photoresist (IP-DIP, Nanoscribe, Germany) and by fine-tuning the laser power. Two successive development baths in PGMEA (propylene glycol monomethyl ether acetate) for 10 min and a consecutive bath in isopropanol for 8 min were chosen. A gentle drying of the structures is achieved by redirecting a stream of N_2_ through a bubbler containing isopropanol.

### Titania Single-Inversion

Woodpile PCs were subsequently infiltrated with TiO_2_ by atomic layer deposition (ALD). Depositions took place at 110 °C in a commercial ALD reactor (Savannah 100 by Cambridge Nanotech, Inc.) operating in exposure mode. A Si wafer was coated simultaneously as reference. Titanium isopropoxide (Sigma-Aldrich, 97% purity) and DI water were used as metal and oxygen source, respectively, and introduced alternately by pneumatic ALD valves. Their respective stainless steel reservoirs were kept at 80 °C and at room temperature. For the deposition, pulse times of 0.05 s and 0.02 s were used for the titanium precursor and oxygen source, respectively, under a carrier gas flow of 5 sccm. The residence time without N2 flow and the purge time under 20 sccm of N_2_ were set to 20 s and 90 s, respectively. Considering a nominal growth per cycle of 0.8 Å, the number of cycles was adjusted to yield partially and completely infiltrated woodpile PCs. A further calcination at 600 °C for 4 hours in a tubular furnace was performed to remove the polymer and to convert the as-deposited amorphous TiO_2_ into the denser polycrystalline anatase phase. A heating ramp of 200 °C per hour was chosen to reduce the thermal deterioration of the structure during the calcination process. Indeed, according to the literature, the deposited films are amorphous at the chosen deposition temperature (<150 °C)^41^. Furthermore, its conversion into anatase after annealing at 500 °C has been already reported^42^,^43^. To confirm once again the phase conversion of the TiO_2_ from amorphous to anatase we carried out X-ray diffraction measurements before and after high temperature treatment shown in the supplemental information (Supplementary Fig. 3). Previous studies have reported n = 2.48 for the real part of the refractive index at 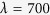 nm for a titania-film deposited under similar conditions. From this we estimate a refractive index of approximately 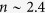 in the near infrared region 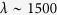 nm studied in our work. For comparison rutile TiO_2_ is birefringent and we can calculate the average refractive index using a weighted sum approach^44^ 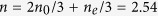 with 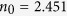 for the ordinary ray and 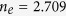 for the extraordinary ray at 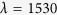 nm (data tabulated in ref. 45). This refractive index value of 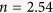 for the rutile phase is consistent with 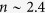 for the anatase phase which is known to be about 5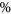 lower.

### Material Characterization

The structure of a direct polymeric and TiO_2_ inverted woodpiles was studied with scanning electron microscopy (SEM), using a Sirion FEG-XL30 S (FEI) microscope working between 5 and 10 kV. Cross-sections were realized by focused ion beam using a Dualbeam NOVA600 Nanolab (FEI). The etching was realized using a Ga ion beam with an acceleration of 30 kV at a current of 3 nA for a couple of minutes followed by a cleaning at 30 kV and 1 nA beam.

The surface roughness and topography of the inverted TiO_2_ woodpile PCs were investigated by atomic force microscopy using a NTEGRA Aura (NT-MDT) in topographic mode with a SKM tip (Supplementary Fig. 1).

### Optical Characterization

Measurements of the optical response of the woodpile PCs were taken using a Fourier transform IR interferometer (Bruker Vertex 70) coupled to a microscope (Bruker Hyperion 2000, liquid N_2_-cooled InSb detector). The objective was a 36× Cassegrain, numerical aperture 0.52. The transmittance of light incident at an angle between 10° and 30° to the surface normal was measured. Spectra were normalized on the same substrate as the residing structures and on a gold mirror for transmittance and reflectance measurements, respectively.

### Numerical calculations of the PCs optical properties

Calculations of the transmittance and reflectance spectra were performed using the FDTD software package MEEP^37^. Band structure calculations were carried out with the freely available MIT photonic bands (MPB) software^46^.

SEM micrographs, Fig. 1d, indicate that the air channels in the inverted and thermally annealed structure show an almost rectangular cross section. Thus for the calculations we assume a rectangular geometry with a height of R_h_ = 415 nm and a width of R_w_ = 243 nm. For the in-plane channel separation we use a = 651 nm and for the inter-plane distance h = 253 nm. As suggested previously^8^, the effect of the presence of voids in the inverted structure can be taken into account by considering an effective refractive index.

To numerically determine the transmission properties of the inverted woodpile, transport simulations were performed with the freely available MEEP software^37^ on a finite stack of a woodpile PC using the same geometrical parameters. The simulated transmission spectra shown in the figures were obtained by calculating the total transmittance of plane waves at normal incidence (Γ − *X*′) (see also Supplementary Fig. 4). The numerical comparison to the experimental data was performed using a cell size of edge length ∼15.6 nm and assuring that the energy is conserved at least within 0.3% in all cases. Matching the position of the gap in the Γ − *X*′ direction with the results obtained in transmission yields an estimated effective refractive index n_eff_ = 2.12 instead of a nominal value of n ≈ 2.4 for bulk TiO_2_ in the anatase phase.

The band structure is calculated based on these assumptions is shown in Fig. 4a. In the inset of Fig. 4b, the path in reciprocal space is shown in detail together with a sketch of the first Brillouin zone of the lattice. As can be seen, the structure corresponds to a slightly stretched Face Center Cubic (FCC) lattice. Indeed, in the current case h/a = 0.388, while in a perfect FCC lattice h/a = 0.353.

To determine the transmission spectrum of the polymer woodpile templates shown in Fig. 2, we assume elliptically shaped rods. For the refractive index of the polymer we assume n = 1.5. (The parameters used in the numerical calculations are adapted for a best fit to the experimental data.) From this we obtain a rod height R_h_ = 343 nm, rod width R_w_ = 323 nm, an in-plane rod separation a = 689 nm and an inter-plane distance h = 267 nm. These lattice parameters are slightly larger, by about 6%, compared to the inverted titania structure. The exact origin of this small difference is not clear as the exact choice of the (best)fitting parameters can be influenced by a number of factors. First, the structure and the optical properties of the material are coupled via the average refractive index of the material. Second, during the inversion and calcination process small geometrical changes of the whole structure but also the rod/channel cross-section can be observed. All of this can have an influence on the optical parameters. Modeling such small imperfections is difficult and beyond the scope of this work.

## Additional Information

**How to cite this article**: Marichy, C. *et al.* High-quality photonic crystals with a nearly complete band gap obtainedby direct inversion of woodpile templates with titanium dioxide. *Sci. Rep.* **6**, 21818; doi: 10.1038/srep21818 (2016).

## Supplementary Material

Supplementary Figures
