# Supplementary material for: High-quality photonic crystals with a nearly complete band gap obtained by direct inversion of woodpile templates with titanium dioxide
Source: Sci Rep. 2016 Feb 25;6:21818. doi: 10.1038/srep21818 (PMC4766404; doi:10.1038/srep21818)
Supplement: Supplementary Figures [file srep21818-s1.pdf]

Supplementary Information: High-quality photonic crystals with a nearly complete band gap obtained by direct inversion of woodpile templates with titanium dioxide

Catherine Marichy,[1] Nicolas Muller,[1] Luis S. Froufe-Pérez,[1] and Frank Scheffold[1,*]

[1] *Department of Physics, University of Fribourg, Chemin du Musée 3, CH-1700, Fribourg, Switzerland*

Material Characterization

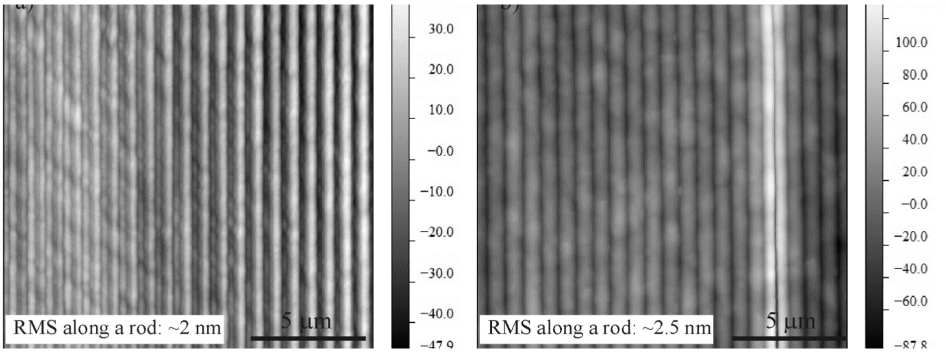

Figure 1: AFM characterizations of a TiO_2 a) hollow-channel and b) inverse woodpile PCs obtained after 1000 and 4500 ALD cycles and subsequent calcination.

In order to confirm the removal of the polymer during the high temperature annealing step, spectra in mid-infrared (MIR) were recorded on woodpiles supported on CaF_2 before and after infiltration and calcination using the same IR microscope also used for the NIR spectroscopy (see Methods). In Figure 2, the typical vibration bands of the polymeric functional groups are visible (grey line). After TiO_2 ALD, additionally to the characteristic signature of the polymer, a strong broad band between 3100 and 3500 cm^{-1} corresponding to the vibrational mode of OH groups (ν_{OH}) can be observed (light grey line). Dashed lines indicate the four principal bands of the polymer, visible on both spectra, before and after infiltration. One can notice the disappearance of nearly all bands after calcination (black line); mainly Fabry-Pérot fringes and a peak around 2400 cm^{-1}, attributed to CO_2, are observed, sign of the polymer removal and TiO_2 densification. The strong CO_2 peak could be attributed to the polymer decomposition; a part of the gas could indeed remain trapped in the 3D structures.

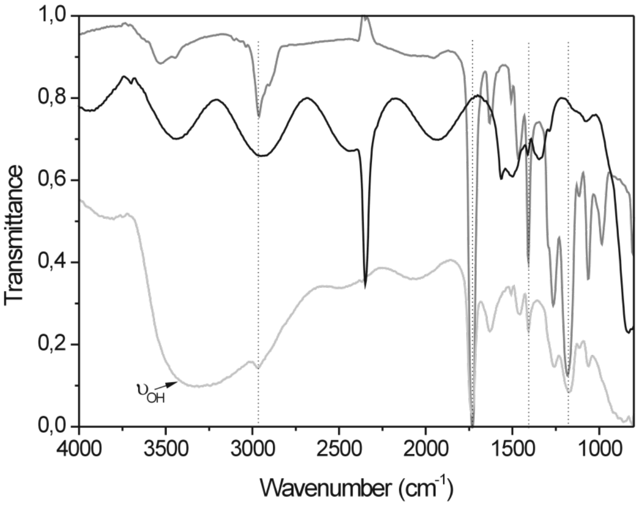

Figure 2: MIR transmittance spectra are shown for polymer (grey line), TiO_2/polymer (light grey line) and TiO_2 (black line) 3D structures supported on CaF_2 glass.

To confirm the phase conversion of the TiO_2 from amorphous to anatase we carried out X-ray diffraction measurements before and after high temperature treatment. X-ray diffraction patterns of as prepared (black line) and heat treated (red line) 100 nm TiO_2 film deposited via ALD on glass are shown in Figure 3. The reference pattern (JCPDS card No. 004-0477) is also added to the figure. The figure shows that as-prepared the titania film is amorphous, while after heat treatment at 600°C, peaks corresponding to the diffraction planes (101) and (200) of the TiO_2 anatase phase appear. The large band visible on both patterns is attributed to the amorphous glass support. Furthermore the thickness of the film explains the small intensity of the observed peaks as well as the presence of the peaks at 25.35 and 38.51° only as they are the most intense for the anatase phase.

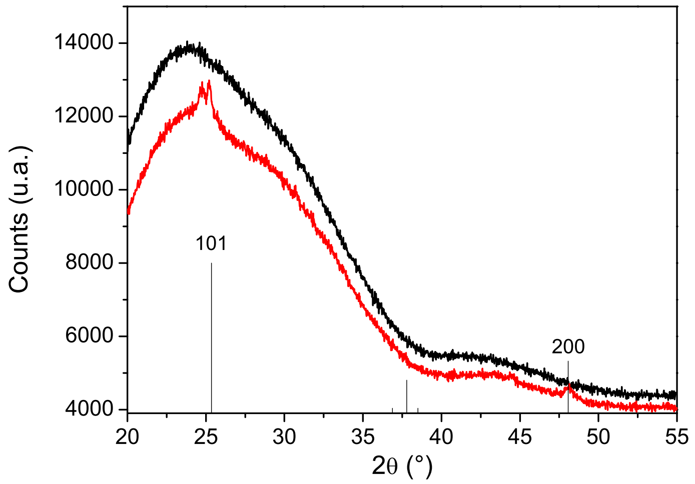

Figure 3: XRD measurements of a 100 nm TiO_2 film deposited via ALD on glass using a PAN Analytical diffractometer working at 45 kV and 30 mA with copper radiation (λ = 1.54 Å). Scans in $\theta - 2\theta$ mode were recorded from 20 to 55° (2θ) with a time per step of 2000 s. Upper curve (black): as-prepared titania film. Lower curve (red): after heat treatment at 600°C.

Transmittance spectra

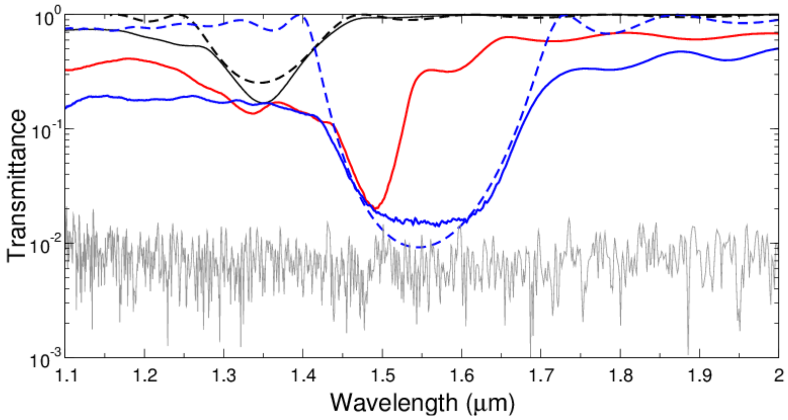

Figure 4: Logarithmic representation of the data shown in Figure 2 a). Transmittance spectra of a polymer template as obtained by DLW (black full line), the partially infiltrated TiO_2 hollow-rod structures (red full line) and the TiO_2 inverted structure (blue full line) showing a nearly complete bandgap. Calculated transmittance spectra at normal incidence are shown as dashed lines. The light gray lines denote the baseline noise indicating the minimum measurable transmittance of the instrument, recorded on a gold mirror.

FDTD calculations

The actual measurements are not performed at normal incidence but using a Cassegrain objective spanning angles between 10° and 30° from the normal. To verify that our calculations at normal incidence accurately reflect the experimental situations we calculate the band structure along a path in the boundary of the effective illumination cone, as shown in Figure 5, after considering the refraction in a medium of effective refractive index n_{eff}=2.12. As shown in the Figure S4, the $\Gamma - X'$ gap (full bandwidth=14.8 %) and the gap found considering all the incoming angles (full bandwidth=12.2 %) differ only slightly in term of width and central position. Hence, it can be concluded that simplified normal incidence simulations provide accurate results for the transport spectra capturing the essential characteristics of the fabricated structures.

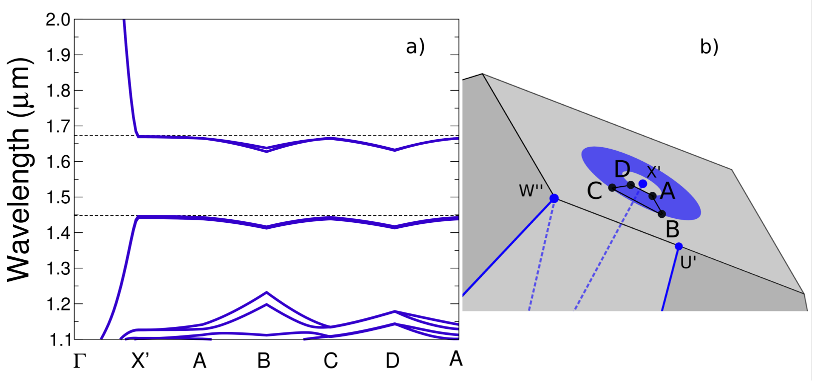

Figure 5: a) Photonic band structure along a path in the first Brillouin zone within the illumination hollow cone given by the Cassegrain objective. The cone minimum and maximum angles are reduced according to the refraction at the boundary between air and the host material with to n_{eff}=2.12 corresponding to the effective permittivity of the TiO_2 with voids. In b) , a detail of the 1st BZ is shown together with the points in the reciprocal space forming the path in a).

* Electronic address: `frank.scheffold@unifr.ch`
